# A NaHCO_3_-Tolerant Endophyte *Bacillus amyloliquefaciens ZmBA DSM7* Enhances Growth and Mitigates NaHCO_3_-Induced Alkaline Stress in Maize Through Multiple Mechanism

**DOI:** 10.3390/plants14243742

**Published:** 2025-12-08

**Authors:** Guoliang Li, Wenhao Wan, Miaoxin Shi, Huitao Cui, Fengshan Yang, Wei Yang, Shumei Jin

**Affiliations:** 1 Heilongjiang Academy of Agricultural Sciences, Harbin 150086, China; 2 Key Laboratory of Saline-Alkali Vegetation Ecology Restoration, Ministry of Education, College of Life Sciences, Northeast Forestry University, Harbin 150040, China; wwh13589976069@163.com (W.W.); shimiaoxin0224@163.com (M.S.); meow0198@163.com (H.C.); jinshumei1972@163.com (S.J.); 3 Engineering Research Center of Agricultural Microbiology Technology, Ministry of Education, Heilongjiang University, Harbin 150080, China; yangfengshan@hlju.edu.cn; 4 Heilongjiang Agricultural Technology Extension Station, Harbin 150090, China; yxwyyy@126.com

**Keywords:** alkaline stress, *Bacillus amyloliquefaciens*, maize, plant growth-promoting, *ZmBA DSM7*

## Abstract

Background Soil alkalization inhibits plant growth and yield, the endophytic plant growth-promoting bacteria (PGPB) can alleviate salt stresses for plant. Methods: Isolate a NaHCO_3_-tolerant *Bacillus amyloliquefaciens* strain (*ZmBA DSM7*), characterize its PGP traits, elucidate the physiological and biochemical mechanisms by which it enhances maize growth. Results: A *ZmBA DSM7* strain was isolated from the root of maize growing in a mildly alkaline soil (pH 8.8). The strain exhibited high tolerance to 500 mM NaHCO_3_ and maintained its PGP traits, *ZmBA DSM7* had a positive effect on maize seed germination and alkaline stress tolerance by enhancing seed vigor under normal or alkaline growth conditions. The maize seedlings inoculation with *ZmBA DSM7* markedly improved chlorophyll content and reduced oxidative damage by lowering malondialdehyde (MDA) content and enhancing the antioxidant enzymes activities in the pots. In the field, *ZmBA DSM7*-inoculated plants showed a increase in yield (such as the ear length, the number of kernels row number, average spike weight, the 100-grain weight, and so on). Conclusion: The *ZmBA DSM7* promotes maize growth and mitigates NaHCO_3_-induced alkaline stress in maize by a multifaceted mechanism involving enhanced nutrient acquisition (N, P and K) and antioxidant status and improved soil quality.

## 1. Introduction

Soil alkalization of Songnen Plain is primarily caused by excess NaHCO_3_ and Na_2_CO_3_ [1], sodic soil induces highly alkaline pH and nutrient deficiencies, it is a significant abiotic stress that severely inhibits plant growth and productivity worldwide [2,3]. Improving plant salt and alkali tolerance is a signifcan issue for agricultural pursuits.

The endophytic PGPB can help host plants absorption nutrients such as nitrogen, phosphorus, and potassium from the soil to promote plant growth and may increase plant growth [4,5,6]. The characteristic of endophytes in enhancing plant growth provides may enhance plant adaptation to environmental stress and provide a new solution to improve the salt tolerance of plants.

Maize is the main grain crop in cereals and accounts for one-third of the total major crop harvest area [6,7]. Maize exhibits severe growth retardation and reduced yield under salt conditions [7], sustainable strategies to mitigate this stress are urgently needed [3,8,9]. The endophytic bacteria from various maize have been shown to promote the growth and development of maize [10,11,12,13,14,15,16,17].

*B. amyloliquefaciens* were isolated from plant-pathogen-infested soil possess plant-growth-promoting activity at first [18,19,20,21]. Some *B. amyloliquefaciens* as rhizosphere microorganisms and some exist inner tissues of plants, becoming endophytes [22,23]. The *B. amyloliquefacien* is important for plant growth stimulation, It can promote plant rooting, increase root vitality, and increase the area of soil nutrient absorption by roots [24,25,26,27,28,29,30,31,32,33]. *B. amyloliquefaciens* had high saline alkali tolerance and growth promotion ability, as well as great application potential for the improvement of saline alkali soil [34,35,36].

*B. amyloliquefaciens* have potential to improve maize growth and yield under stress condition [37,38,39,40]. But in-depth studies on endophytic *B. amyloliquefaciens* from maize under alkaline stress were limited. A endogenous *B. amyloliquefaciens* strain was isolated from maize in pH 8.8 soil. This bacterium has strong saline-alkali tolerance, promotes plant growth, and improves soil pH. The search for sustainable agriculture has increased interest in using endophytic bacteria to reduce fertilizer use and increase stress resilience.

## 2. Results

### 2.1. Isolation and Identification of a NaHCO_3_-Tolerant Bacterium

#### 2.1.1. Isolation of a NaHCO_3_-Tolerant Bacterium

A bacterial isolate was obtained from the NA solid medium added with 500 mM NaHCO_3_, the colony that can grow on 500 mM NaHCO_3_ medium indicates that the colony has strong salt alkali resistance.

#### 2.1.2. Characterization and Sequencing Analysis of a NaHCO_3_-Tolerant Bacterium

**Figure 1:**
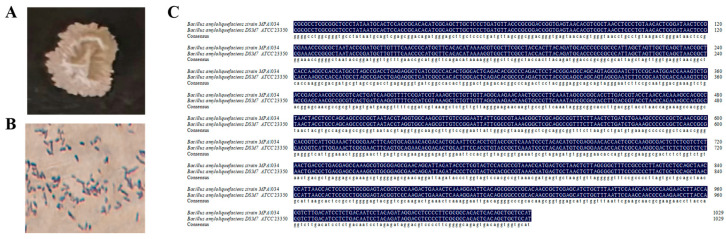
Colonies grown in the NA medium and blasted with standard bacterial strain in the BERGEY’S MANUAL. (**A**) Colonies grown in the NA medium. (**B**) Colonies stained with Gram. (**C**) Blast with standard bacterial strain in BERGEY’S MANUAL.

The *B. amyloliquefaciens* strain grows well on NA plate medium. The colonies of the strain can form nearly circular or irregularly shaped colonies, which are milky white and opaque, with a dry surface, raised edges, uneven colony edges, wrinkles on the surface with irregular margin, and no luster Figure 1A). It does not produce pigments, gram positive, and exhibits a straight rod-shaped morphology (Figure 1B).

Blast the 16S rRNA sequence of the bacterial isolate using the NCBI database. Most of the Blast result belongs to the genus of *B. amyloliquefaciens* type strain. It is 100% identical to the *B. amyloliquefaciens* strain DSM7 (NCBI reference sequence NR_118950.1) [23] (Figure 1C). The strain was identified as *B. amyloliquefaciens* strain DSM 7. Because it is an endophyte extracted from *Zea mays* L., it is named *ZmBA DSM7* DSM7 in this study.

#### 2.1.3. Assay Physiological and Biochemical Identification of ZmBA DSM7

**Figure 2:**
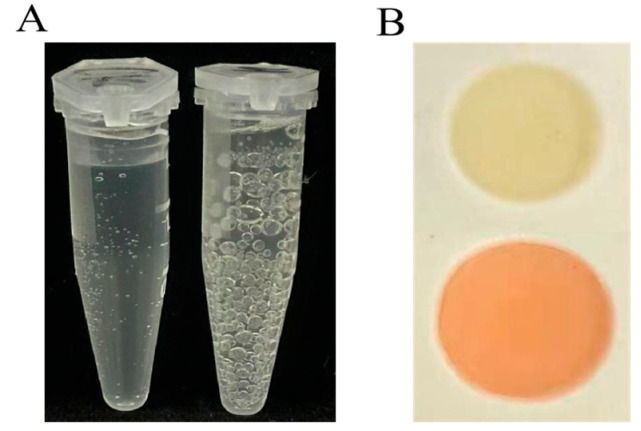
Physiological and biochemical reactions of bacteria. (**A**) Catalase characteristic experiment for catalase positive or negative. (**B**) Voges–Proskauer (VP) test for producing acid reaction.

The bubbles appear in the catalase characteristic experiment within half a minute, indicating acatalase positive result (Figure 2A). The bacterium produced a target organic compound in the Voges-Proskauer (VP) test, showing the ability (Figure 2B). These two features are consistent with the physiological characteristics of *B. amyloliquefaciens* in the Berger Handbook.

#### 2.1.4. Analysis of the Growth-Promoting Characteristics of ZmBA DSM7

**Figure 3:**
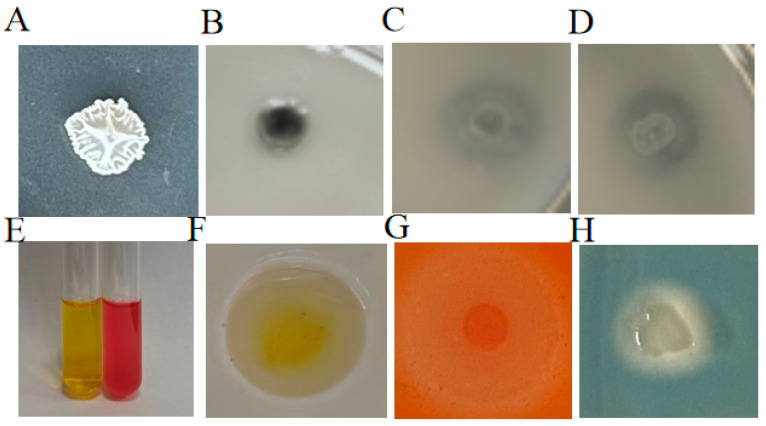
Schematic illustration showing the various tests performed on plates (Petri dishes 9 cm in diameter) for the assessment of the PGP traits of *B. amyloliquefaciens*. (**A**) The capacity to produce ACC deaminase was measured on ADF medium; (**B**) Nitrogen fixation ability on the Ashby culture medium; (**C**) phosphorus solubilization ability on the NBRIP culture medium; (**D**) The capacity to decompose potassium was measured on silicate medium; (**E**) The capacity to produce IAA was measured used Salkowski’s reaction; (**F**) The capacity of ammonia production on the Nessler’s reagent culture medium; (**G**) The ability to dissolve cellulose was assessed on CDMM medium; (**H**) Siderophore production on the CAS culture medium.

*ZmBA DSM7* is capable of growing on ADF medium with ACC serving as the sole nitrogen source (Figure 3A). *ZmBA DSM7* has nitrogen fixation ability because the *ZmBA DSM7* can grow normally in an Ashby nitrogen-free culture medium (Figure 3B). A transparent circle around the *ZmBA DSM7* colony on a NBRIP phosphorus solubilized, and solubilize potassium medium, this showed the strain has phosphorus solubilization and solubilize potassium ability (Figure 3C,D). The LB culture medium containing the *ZmBA DSM7* exhibited red with the control group, thereby substantiating the production of IAA (Figure 3E). *ZmBA DSM7* is capable of ammonia production because a yellow precipitate appeared on the Nessler’s reagent culture medium (Figure 3F). An orange circle is produced around the bacterial colony indicaing of dissolving cellulose and siderophore production by the bacterium (Figure 3G,H).

#### 2.1.5. The ZmBA DSM7 Growth-Promoting Traits

##### Growth of *ZmBA DSM7* Under NaHCO_3_ Stress

**Figure 4:**
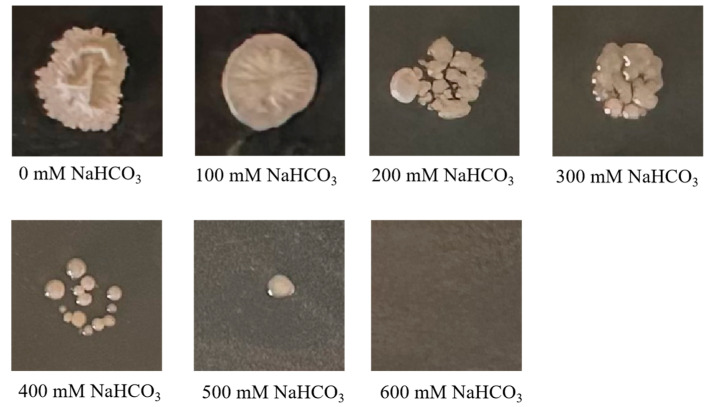
A total of 5 µL *ZmBA DSM7* (OD_600_ = 0.5) were spotted on solid LB media supplemented with different NaHCO_3_ content stresses and grew at 30 °C for 3 days.

The *ZmBA DSM7* isolates were able to grow on NA medium with 0–500 mM NaHCO_3_ (Figure 4). As the concentration of NaHCO_3_ stress increases, bacterial colonies showed reduced growth, when the concentration was increased to 600 mM NaHCO_3_, no colony growth on culture medium. *ZmBA DSM7* exhibited remarkable tolerance, growing optimally in up to 500 mM NaHCO_3_.

##### Growth of *ZmBA DSM7* Under Different pH Value

**Figure 5:**
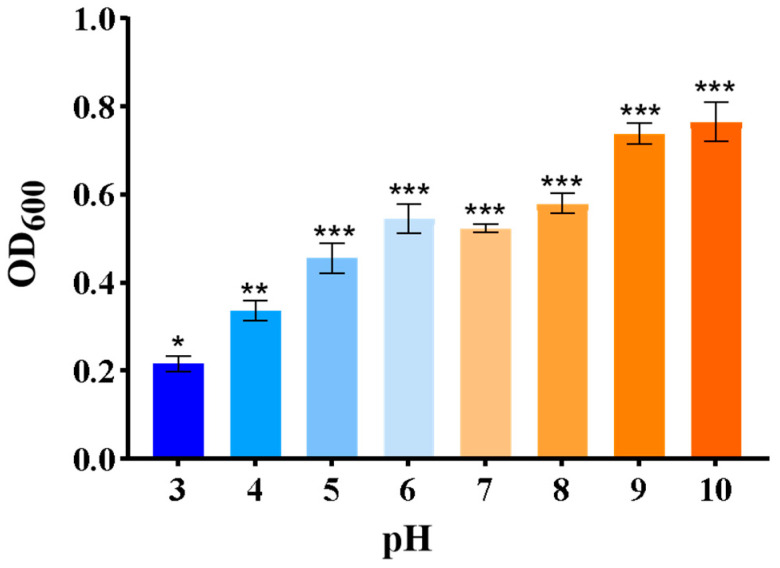
Growth of *ZmBA DSM7* under different pH conditions. A total of 200 µL *B. amyloliquefaciens* (OD_600_ = 0.5) liquid was added to 1 mL sterilized LB liquid medium with different pH values (3–10) at 30 °C with constant shaking at 130 rpm. The OD_600_ value was measured using an ELISA reader every and measured continuously for 24 h. * *p* < 0.05, ** *p* < 0.01, *** *p* < 0.001, standard error of three biological replicates.

The *B. amyloliquefaciens* bacterium can grow at the pH values of 9–10 and be inhibited in growth at pH 3 (Figure 5), This indicates that the bacteria *ZmBA DSM7* have high pH tolerance and prefer alkaline condition.

### 2.2. Effect of ZmBA DSM7 on Growth of Maize

#### 2.2.1. ZmBA DSM7 Effect on Maize Seeds Germination

**Figure 6:**
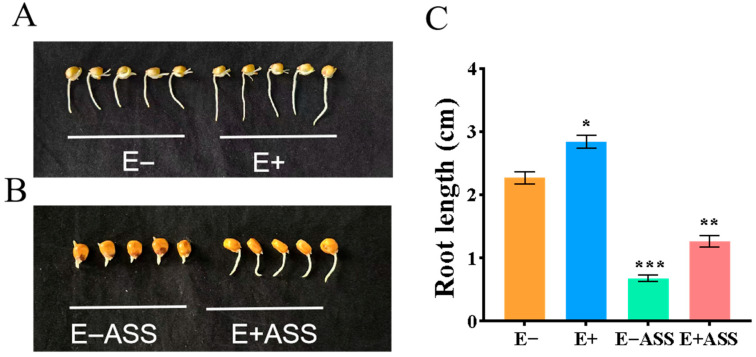
The effect of *ZmBA DSM7* on maize seed germination. (**A**) E−: the sterile maize seeds were planted on the filter paper; E+: the sterile maize seeds in the inoculation with *ZmBA DSM7* were planted on the filter paper; (**B**) E−ASS: the sterile maize seeds were planted on the filter paper + 100 mM NaHCO_3_; E+ASS: sterile maize seeds in the inoculation with *ZmBA DSM7* were planted on the filter paper + 100 mM NaHCO_3_; (**C**) The root length of maize seed germination for 10 days. Data show the means *±* SE of three replicates. At least 50 seeds in each treatment were measured in each repeat. * *p* < 0.05, ** *p* < 0.01, *** *p* < 0.001, standard error of three biological replicates.

Seed germination rate all are 100% in this experiment, the maize seed inoculated with *ZmBA DSM7* germination (germination in 3 days) was faster than that in the control group (germination in 4 days). The germination of maize seeds was inhibited under the stress treatment, the maize seed inoculated with *ZmBA DSM7* germination (germination in 5 days) was faster than that in the control group (germination in 6 days) under the 100 mM NaHCO_3_ stress treatment. The inoculation group roots also grew longer than the control regardless of the presence or absence of stress conditions (Figure 6).

**Figure 7:**
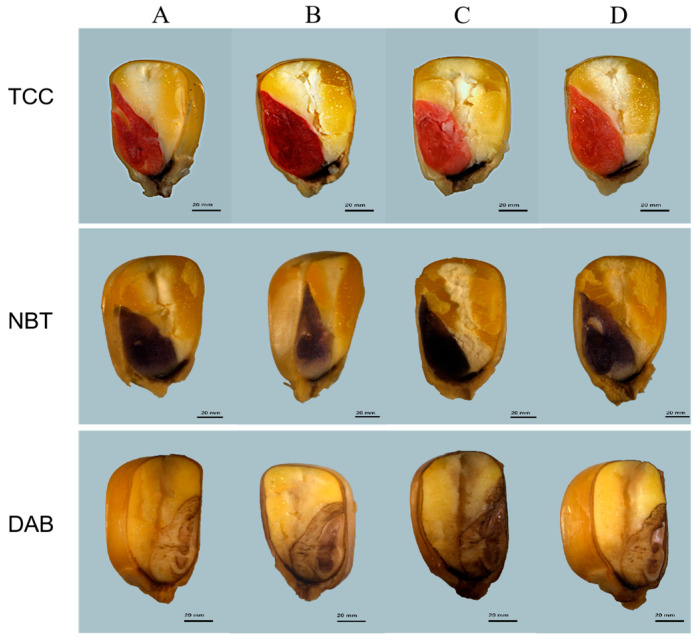
TTC, NBT and DAB staining for maize seed vigor and ROS content. Each group A represents the sterile maize seeds were planted on the filter pape; Each group B represents the sterile maize seeds in the inoculation with *ZmBA DSM7* under free salt stress; Each group C represents the sterile maize seeds were planted on the filter paper+100 mM NaHCO_3_; Each group D sterile maize seeds in the inoculation with *ZmBA DSM7* were planted on the filter paper+10 mM NaHCO_3_.

The top group is measured maize seed vigor using TTC staining; the maize seed embryos were stain deep red under free salt stress and light red under the 100 mM NaHCO_3_ stress, but those the maize seed embryos inoculated with *ZmBA DSM7* were darker than those without inoculation under NaHCO_3_ stress. The middle group is measured maize seed dynamic accumulation of O_2_^−^ using the NBT staining; The bottom group is measured maize seed dynamic accumulation of H_2_O_2_ using the DAB staining; The seed embryos were stained light blue or light brown under free salt stress and darker brown under the 100 mM NaHCO_3_ stress, but those the maize seed embryos inoculated with *ZmBA DSM7* were lighter than those without inoculation under NaHCO_3_ stress (Figure 7). So, *ZmBA DSM7* is beneficial for the germination of maize.

#### 2.2.2. The Effect of ZmBA DSM7 on the Maize Seedlings in the Pots

##### *B. amyloliquefaciens* Bacterial Strains to Colonize Maize Seedling

**Figure 8:**
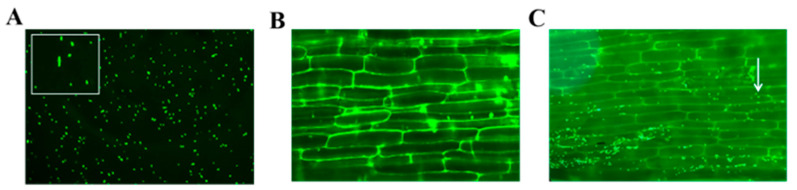
*ZmBA DSM7* with GFP colonization in maize roots. (**A**) The morphology of GFP-expressing *ZmBA DSM7*; (**B**) the maize root without inoculation with GFP-expressing *ZmBA DSM7* as a control; (**C**) the colonization of maize root with GFP- expressing *ZmBA DSM7*. The white arrow points to a bacterial cluster, scale Bar = 50 µm.

To assess the ability of the bacterial strains to colonize maize seedling. Transform GFP protein into *ZmBA DSM7*, the GFP fluorescence signal remained almost unchanged over time, indicating that the strain is highly stable. Labeled *ZmBA DSM7* with GFP colonization in maize roots were observed using microscopy (Figure 8).

##### The Effect of *ZmBA DSM7* on the Maize Seedling in the Pots

**Figure 9:**
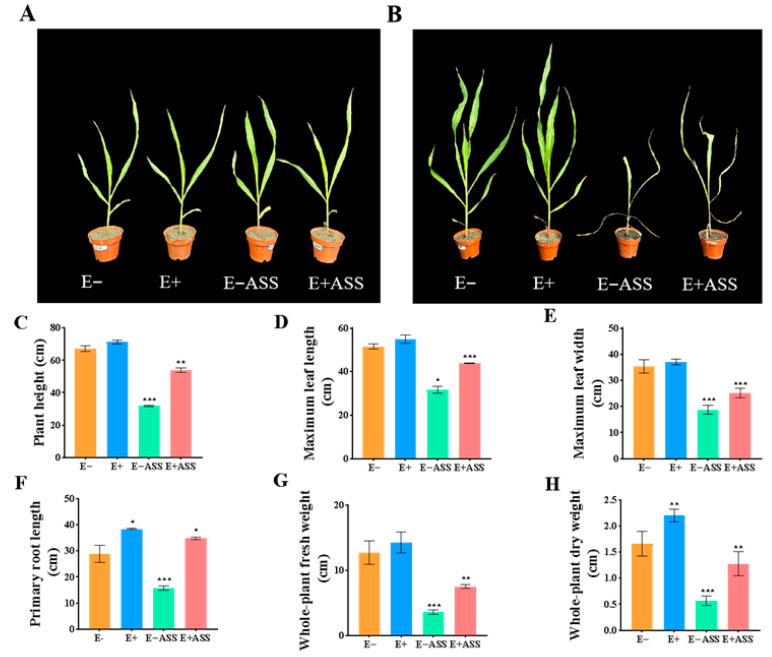
Effect of *ZmBA DSM7* strain on maize growth parameters after cultivation 7 days under normal or saline conditions. (**A**) Phenotype of seedlings before treatment; (**B**) Phenotype of seedlings after treatment. The growth of maize seedling, from leaf to right: uninoculated maize seedling growth under free salt, inoculated maize seedling with *ZmBA DSM7*, uninoculated maize seedling growth under NaHCO_3_ stress, and inoculated maize seedling with *ZmBA DSM7* under NaHCO_3_ stress; (**C**) Plant height; (**D**) Maximum leaf length; (**E**) Maximum leaf width; (**F**) primary root length; (**G**) whole plant fresh weight; (**H**) whole plant dry weight, E− represents uninoculated maize seedlings. E+ represents inoculated maize seedlings, E−ASS represents uninoculated maize seedlings irrigated with 400 mM NaHCO_3_, and E+ASS represents inoculated maize seedlings irrigated with 400 mM NaHCO_3_. The values represent the means of replicates (n = 4) ± standard deviations. Asterisks in superscript indicate a significant difference from the control at 95% between treatments. * *p* < 0.05, ** *p* < 0.01, *** *p* < 0.001. Each data point is the average of five replicates, and error bars represent.

To characterize the effect of *ZmBA DSM7* on the maize seedling, the *ZmBA DSM7* strain promoted plant growth in the absence as well as in the presence of salt stress(Figure 9A,B). At the jointing stage, compared with the CK group, the inoculated maize plants showed remarkable improvements in key growth indicators. The plant height (Figure 9C), leaf length (Figure 9D), leaf width (Figure 9E) root length (Figure 9F), plant fresh weight (Figure 9G) and plant dry weight (Figure 9H) were increased, NaHCO_3_ stress severely stunted the growth of non-inoculated maize plants, the endophytic bacteria inoculation effectively alleviated the damage caused by salt stress.

**Figure 10:**
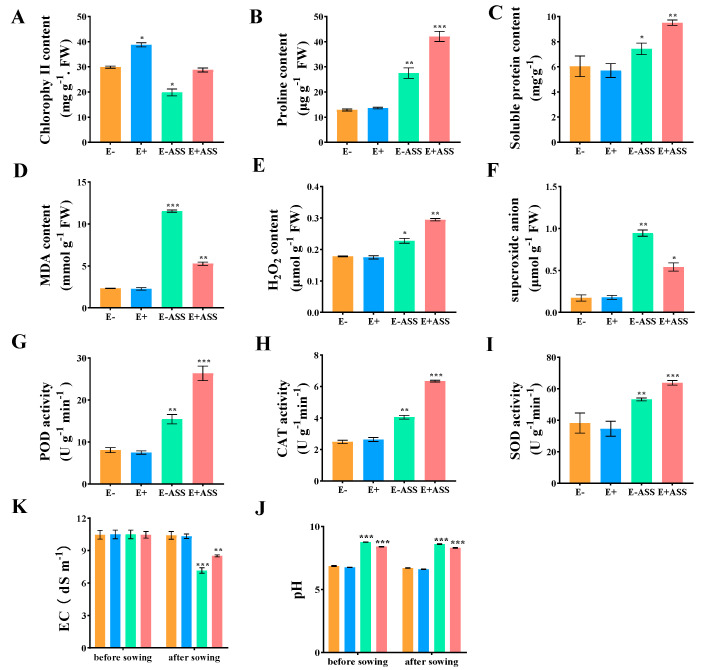
Antioxidant enzyme activity determination in maize seeding. (**A**) H_2_O_2_ content. (**B**) proline content; (**C**) MDA content; (**D**) soluble protein content; (**E**) POD activity; (**F**) CAT activity; (**G**) chlorophy II content; (**H**) SOD activity; (**I**) superoxide anion; (**K**) EC; (**J**) pH value. * Significance at *p* < 0.05, ** Significance *p* < 0.01, *** Significance *p* < 0.001.

The chlorophyll SPAD value was higher than that of CK (Figure 10A), indicating a stronger photosynthetic capacity. Meanwhile, the proline content (Figure 10B) and soluble protein (Figure 10C) were increased than the CK. MDA content in the leaves was reduced compared with CK (Figure 10D). H_2_O_2_ content (Figure 10E) and superoxide anion (Figure 10F) were increased than the CK. Some antioxidant enzyme activity such as POD (Figure 10G), CAT (Figure 10F), and SOD (Figure 10H) increased. Both the soil electrical conductivity (Figure 10K) and pH (Figure 10J) value decreased compared with before sowing. These changes indicated that *ZmBA DSM7* inoculation helped maintain the integrity of the maize cell membrane and enhanced the osmotic regulation ability and antioxidant capacity and improved the soil saline environment, alleviates growth inhibition under NaHCO_3_ stress.

#### 2.2.3. The Effect of ZmBA DSM7 on the Maize Seedling in the Filds

**Figure 11:**
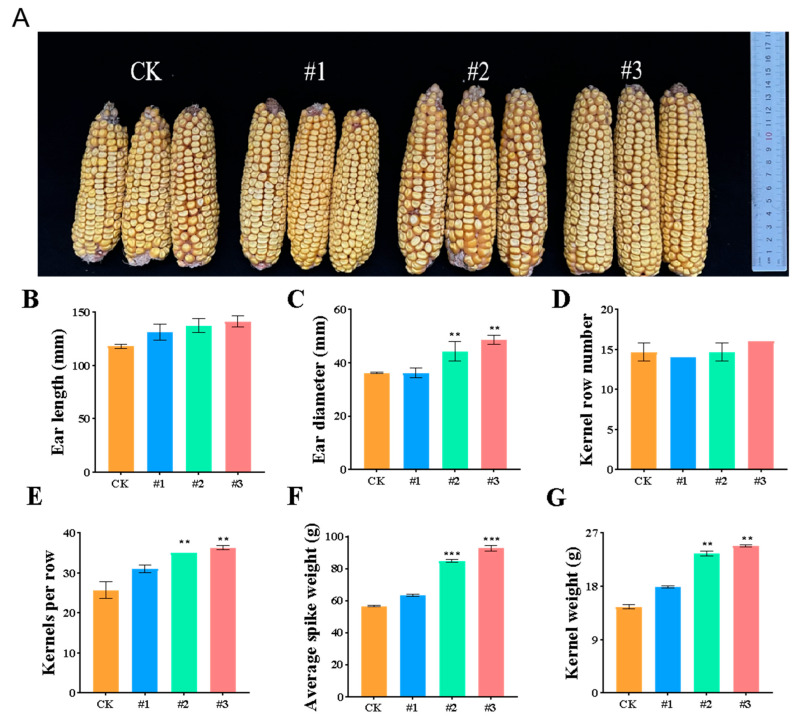
Effects of different treatments on maize yield in the normal field. (**A**) Phynotype of maize corncobs collected during the maturation stage; (**B**) Ear length; (**C**) Ear diameter; (**D**) Kernel row number; (**E**) Kernels per row; (**F**) average spike weight; (**G**) Kernel weight. CK: the control group was soaked in sterile water #1: maize seeds were immersed in a *ZmBA DSM7* suspension. #2: the seedling irragated with equal volume water. #3: the seedling irragated with the *ZmBA DSM7*. ** *p* < 0.01 and *** *p* < 0.001, standard error of three biological replicates.

At the maturity stage, the yield-related traits of maize were also significantly improved by *ZmBA DSM7* (Figure 11A). The ear length (Figure 11B), ear diameter (Figure 11C), was longer than that of CK, the number of kernels row number (Figure 11D), kernels per row (Figure 11E) and average spike weight (Figure 11F), Kernel weight (Figure 11G). The grain yield were higher than the CK group (Figure 11). *ZmBA DSM7* improve maize growth in field experiments.

##### Endophytic Bacteria Improve Maize Salt Tolerance in Field Experiments

**Figure 12:**
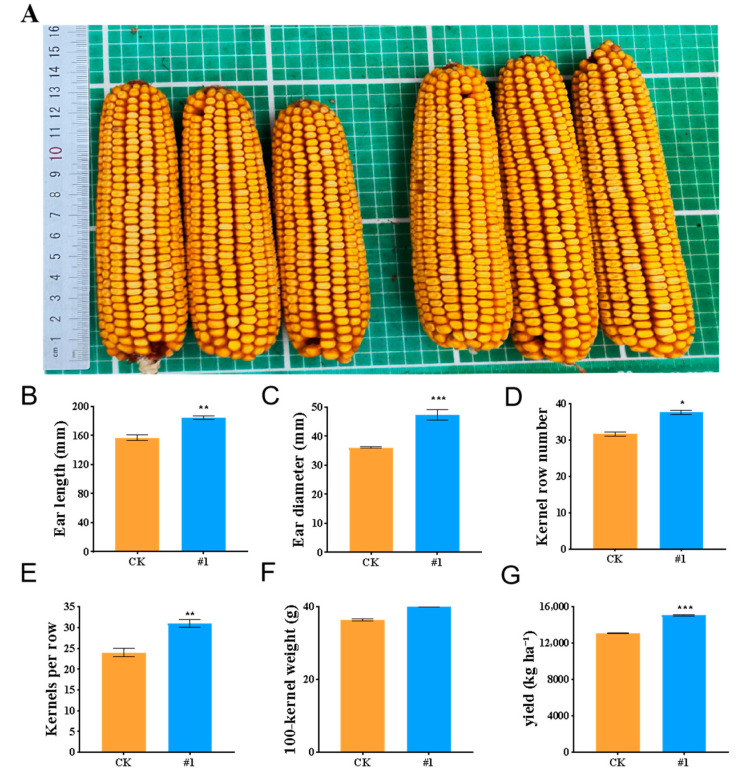
Yield index of maize based on the seedling irragated with the *ZmBA DSM7* under salt field (**A**) Phynotype of maize corncobs collected during the maturation stage left: the control group was soaked in sterile water. right: the seedling irragated with the *ZmBA DSM7;* (**B**) Ear length; (**C**) Ear diameter; (**D**) Kernel row number; (**E**) Kernels per row; (**F**) 100 spike weight; (**G**) yield. CK: the control group was irragated with water. #1: the seedling irragated with the *ZmBA DSM7*. * *p* < 0.05, ** *p* < 0.01 and *** *p* < 0.001, standard error of three biological replicates.

In the alkali soil, the yield-related traits of maize were also significantly improved by *ZmBA DSM7* (Figure 12A). The ear length (Figure 12B), ear diameter (Figure 12C), was longer than that of CK, the number of kernels row number (Figure 12D), kernels per row (Figure 12E), 100-kernel weight (Figure 12F). and yield (Figure 12G) were higher than the CK group. This field experiment fully demonstrated that endophytic bacteria can be used as an effective biological measure to improve the salt tolerance and yield of maize in saline—alkali farm land.

**Figure 13:**
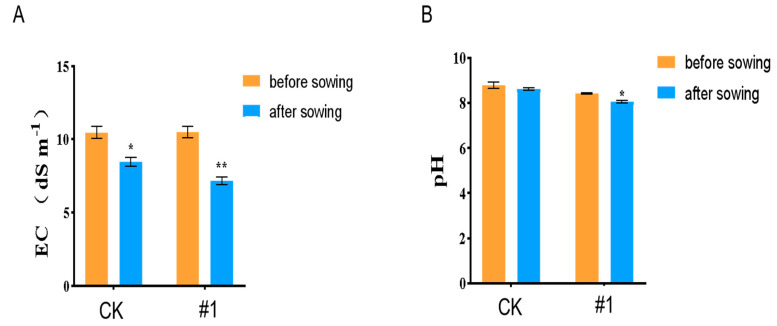
*ZmBA DSM7* strain effect EC and pH value in soil pH. (**A**) *ZmBA DSM7* strain effect EC in the soil; (**B**) *ZmBA DSM7* strain effect pH value in the soil. * *p* < 0.05, ** *p* < 0.01, standard error of three biological replicates.

Further analysis of soil samples showed that after harvest, the soil electrical conductivity (Figure 13A) and pH value (Figure 13B) decreased compared with before sowing, this suggesting that endophytic bacteria might also play a role in improving the soil saline environment while enhancing maize salt tolerance.

## 3. Discussion

*Bacillus amyloliquefaciens* exist in both rhizosphere soil [41,42] and plant tissues [43,44]. At present, research and application of *B. amyloliquefaciens* are mainly focused on biocontrol bacteria [34,45,46,47]. The most *B. amyloliquefaciens* strains tolerate 10% NaCl [23]. *B. amyloliquefaciens* were screened from the maize [24], but no in-depth research has been conducted. A endophytic *B. amyloliquefaciens ZmBA DSM7* was isolated from the roots of maize grown in saline–alkali soil with a pH value of 8.8 (Figure 1).

ZmBA DSM7 is catalase positive strain (Figure 2), this bacterium colonizes plants, increasing the activity of cas in the plant body. Which can improve the plant’s ability to resist salt and alkali. Under abiotic stress, seed germination and root growth are adversely affected due to high level of ethylene production [38,48,49]. The secretion of the plant growth hormone auxin, such as IAA, biological nitrogen fixation, synthesis and release of phytohormones, phosphate solubilization, that promote plant growth directly or indirectly [40,50,51,52,53,54,55,56,57,58]. A series of studies has elucidated *B. amyloliquefaciens* of its plant growth-promoting activity (Figure 3).

*ZmBA DSM7* were able to grow normally under 500 mM NaHCO_3_ (Figure 4) or pH value from 5 to 10 (Figure 5), this result aligns with the optimum pH range of 9–10 for the growth of *B. amyloliquefaciens* as stated in the Manual of Systematic Bacteriology [23], After infecting maize seeds with *ZmBA DSM7*, *ZmBA DSM7* can promote the germination of maize and increase the length of maize roots (Figure 6) and improve seed vigor (Figure 7). It indicating that the bacteria can survive and function under alkali conditions with high-pH environments.

*B. amyloliquefaciens* is an excellent tool for studying plant colonization under competitive, environmental conditions; [59,60,61], the *gfp*-tagged strain could be detected in roots tissues of maize (Figure 8), this indicated that *ZmBA DSM7* owns a good transduction in host plant. *ZmBA DSM7* promoted maize root length and plant dry weight (Figure 9),plant height is a widely recognized indicator of overall plant vigor and health status [11,62]. Taller plants after irragated by *B.Amyloliquefaciens ZmBA DSM7* demonstrate a robust ability to utilize available nutrients and water efficiently, which contributes to their vigorous growth.

The maize seedlings inoculation with *ZmBA DSM7* markedly improved chlorophyll content, *ZmBA DSM7* reduced oxidative damage by lowering MDA content and enhancing the antioxidant enzymes activities (Figure 10). This showed *ZmBA DSM7* helps scavenge reactive oxygen species (ROS) by activating a more efficient antioxidant system, preventing cellular damage and maintaining membrane integrity (lower MDA). Catalase strengthening the host’s antioxidant defense system.

A significant improvement in the plant height, cob length, number of grains cob, grain yield and straw yield also justified the imperative role of combined organic and inorganic fertilizers along with the PGPR strains [11,63]. *B. Amyloliquefaciens ZmBA DSM7* has potential to improve maize growth and yield under stresses in the field experiment (Figure 11) that demonstrates the practical potential of endophytic bacteria as cost-effective, eco-friendly bio-inoculants for saline-alkali maize production.

The novelty of this study lies in its validation of these effects in a real-world field setting with naturally occurring salt and alkali stresses (soil EC:10.1 dS·m^−1^, pH: 8.8). The *B. Amyloliquefaciens ZmBA DSM7* strain reduction electrical conductivity and slight decrease in soil pH in inoculated treatments (Figure 12). *ZmBA DSM7* may modulate soil alkalinity, a key constraint in saline-alkali lands.

**Figure 14:**
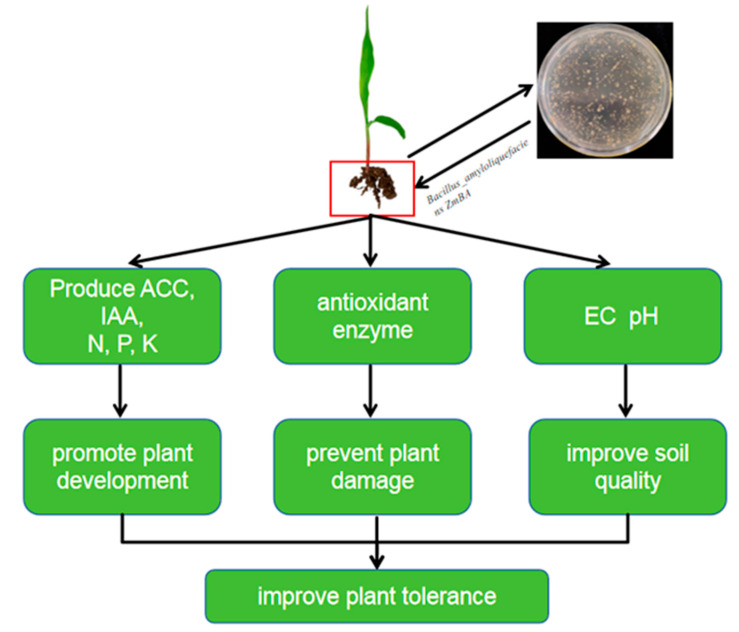
A model for the mechanism underlying *ZmBA DSM7* promoted maize development. The most significant mechanism by which *ZmBA DSM7* mitigates stress as following: Under abiotic stress, production of ACC and ethylene is enhanced in plants, ACC is a precursor for producing ethylene and root growth are adversely affected due to high level of ethylene production, The ACC-deaminase activity of *ZmBA DSM7* strains probably suppressed stress induced ethylene level. The high pH of alkaline soil lack of N and K, andimmobilizes P, *ZmBA DSM7* has the functions of dissolving phosphorus, fixing nitrogen, and dissolving potassium, which can improve nutrient availability (N, P, K) and enhance plant salt tolerance; *ZmBA DSM7* increases antioxidant enzyme activity (CAT, POD, SOD) to reduce oxidative damage and enhance antioxidant defense, enhance plant salt tolerance; After irrigating the soil with *ZmBA DSM7*, reduction electrical conductivity (EC) and decrease in soil pH in inoculated treatments, improving soil quality is beneficial for plant growth.

*B. Amyloliquefaciens ZmBA DSM7* dual benefit in improving both plant tolerance and soil quality. The most significant mechanism by which *B. Amyloliquefaciens ZmBA DSM7* mitigates stress is likely through improving nutrient availability and antioxidant enzyme activity and soil quality (Figure 14). Endophytes offer a sustainable solution by leveraging natural plant-microbe symbioses.

There have a less of potential limitations of this study, controlled-growth chamber often use controlled salt concentrations and ideal growing conditions, the environmental conditions in the field are uncontrollable. This field experiment was only conducted for two years and lacks long-term persistence data, the other soil biological parameters (such as N,P,K content) need to measure. *ZmBA* possible interactions with native soil microbiota that might influence *ZmBA* performance need toverify. This bacterium can lower EC and pH, possibly due to the generation of organic acids, enhanced ion absorption, and changes in rhizosphere chemical properties, more specific molecular mechanisms need to be studied.

In the future, observe whether this strain can promote the growth of other economic crops and improve crop salt-alkali tolerance, replace chemical fertilizers with this bacterium and develop a new type of biological microbial fertilizer.

## 4. Materials and Methods

### 4.1. Isolation and Identification of a NaHCO_3_-Tolerant Bacterium

#### 4.1.1. Isolation of a NaHCO_3_-Tolerant Bacterium

Endophytic bacterial strain were selected from the roots of maize (Longdan181, Salt tolerant variety) grown in saline–alkali soil with pH 8.8 on the DaQing city of Heilongjiang Province in China (Longitude 125.3721239, Latitude: 46.4537278). The isolation method of bacteria is the same as the previous article [64], a endophytic bacterial strain was isolated on NA medium supplemented with 500 mM NaHCO_3_.

#### 4.1.2. Identification of a NaHCO_3_-Tolerant Bacterium

##### Sequencing Analysis of the 16S rRNA Gene

A robustly growing strain was selected. Identification was performed via 16S rRNA gene sequencing (PCR amplification with the primer 27F 5′-AGAGTTTGATCCTGGCTAG-3′ and 1492R 5′-CTACGGCTACCTTGTTACGA-3′, the amplified PCR product 1540 bp), the PCR product was commercially sequenced (Kumei Biotechnology Co., LTD, Changchun, China) and the sequenced results comparing in the GenBank database using the BLAST algorithm (http://www.ncbi.nlm.nih.gov).

### 4.2. The Bacterial Growth-Promoting Traits Under the Normal and Alkaline Stress

The ACC deaminase was determined using ADF (ACC nitrogen source) media and ashby nitrogen-free medium was used for testing the ability of bacterial strain to fix nitrogen fixation capacity, inoculate 2 µL of activated bacterial solution onto a culture medium plate at 30 °C for 24 h [65]; The presence or absence of inorganic phosphorus solubilizing ability of bacteria was tested by inoculating the bacterial isolates in NBRIP (The National Botanical Research Institute’s phosphate) culture medium according to Mehta S, inoculate 2 µL of activated bacteria onto a NBRIP plate at 30 °C for 24 h [66]. The solubilize potassium ability was tested using a silicate medium, inoculate 2 µL of activated bacteria onto a silicate plate at 30 °C for 24 h [32]. The production capacity of IAA was detected by the describe in Bric et al., add 40 mL of the 2.5 mg/mL tryptophan solution into 160 mL DF liquid culture medium. Inoculate the activated bacteria into the above culture medium at 30 °C and 135 rpm for 24 h, take 2 mL of supernatant, and add 50 µL of 50% orthophosphate solution and 4 mL of Salkowski reagent [67]. The capacity of ammonia production on the Nessler’s reagent culture medium, cellulose decomposing microorganism medium for detecting cellulose decomposition, iron carrier (Siderophore) generating capacity was detected on CAS (Chromogenic Agar for Spore) solid medium, Inoculate 2 µL of the strain on a CAS plate. Incubate at 30 °C for 3 days [68].

### 4.3. In Vitro Stress-Tolerance Estimation for Bacterium Under Alkaline Stress

To assess the isolated strain NaHCO_3_ tolerance, 5 µL strain (OD_600_ = 0.5) were grown on solid LB medium plates with 0 (pH 5.80), 100 mM (pH 7.86), 200 mM (pH 7.99) 300 mM (pH 8.03), 400 mM (pH 8.09), 500 mM (pH 8.11), 600 mM (pH 8.23) NaHCO_3_ for 48 h at 37 °C respectively.

To estimate the maximal growth and minimal inhibitory pH value, 5 µL strain was added in liquid LB medium with different pH values (3–10, the pH value of LB liquid culture medium is approximately 5.8. Use NaOH to increase the pH of the culture medium, and use HCL solution to decrease the pH value of the culture medium) and cultivated in a shaker (130 rpm) at 37 °C. The OD_600_ absorbance value was measured using an enzyme-linked immunosorbent assay (ELISA) reader every hour and measured continuously for 24 h.

### 4.4. Plant Growth Promotion and Alkaline Stress

#### 4.4.1. ZmBA DSM7 Effect on Maize Seed Germination

Maize seeds were sterilized and divided into four groups cultivated on filter paper. One group seeds were soaked with H_2_O for 6 h; One group seeds were soaked with *ZmBA DSM7* for 6 h; the third group seeds were treated with H_2_O for 6 h first, followed by 100 mM NaHCO_3_ stress for 30 min; and the last group seeds were treated with *ZmBA DSM7* soaking for 6 h first, followed by 100 mM NaHCO_3_ stress for 30 min. The maize seeds were immersed in different solutions using TTC (2, 3, 5- triphenyltetrazolium chloride) staining(red pigment) for testing seed vigor or DAB (diaminobenzidine) staining (brown pigment) and NBT (nitroblue tetrazolium) staining (blue pigment) for ROS content.

#### 4.4.2. ZmBA DSM7 Effect on Maize Seedling

##### Colonization of *ZmBA DSM7* in Plants

The maize seeds were sown in pots and placed in a controlled-growth chamber at 25 ± 2 °C with a 16 h light/8 h dark cycle. The construction of *ZmBA DSM7* labeled with GFP referred to Lifeng Guo [69]. The *ZmBA DSM7* strain was initially processed to generate competent cells, followed by the delivery of GFP-tagged plasmids (pBBR1MCS2-pAmp-EGFP) into the aforementioned competent cells via electroporation. Finally, the bacterial suspension was plated on LB plates with kanamycin resistance and incubated at 37 °C overnight to screen for the transformants containing pBBR1MCS2. To assess the stability of the GFP fluorescence signal, we examined the strain under a Zeiss fluorescence microscope at daily intervals from day 1 to day 7 post-inoculation. The four-leaf stage maize seedlings in the pots were irrigated with the 50 mL GFP-labeled *ZmBA DSM7* solution for 7 days, and the maize root was visualized by a confocal scanning laser microscope (CSLM) to prove that *ZmBA DSM7* can colonize the maize roots.

##### *ZmBA DSM7* Effect on Maize Seedling

The maize were grown in a greenhouse and subjected to different treatment at the four-leaf stage. The maize seedlings were watered with four irrigation treatments. The first group plants were irrigated only with 50 mL water (as the control); the second group plants were irrigated with the 50 mL resuspended *ZmBA DSM7* solution; the third group plants were irrigated with the 50 mL 400 mM NaHCO_3_ solution after irrigating with 50 mL water for 5 days; and the fourth group plants were irrigated with the 50 mL 400 mM NaHCO_3_ solution after irrigating with 50 mL *ZmBA DSM7* solution for 5 days to test *ZmBA DSM7’s* effect on maize seedlings.

Four irrigation, the leaf chlorophyll content was determined using chlorophyll content meter (Hansatech instruments, Model CL-01). Leave and root lengths were assessed using a ruler, and the dry weights (DW) of the roots and leaves were measured after placing in an oven for 72 h. The activities of Malondialdehyde (MDA), superoxide dismutase (SOD), peroxidase (POD), and catalase (CAT) were assayed spectrophotometrically after treatments for 20 days.

#### 4.4.3. ZmBA DSM7 Effect on Maize Development in the Open Field Tests

##### PGP of Maize in Open Field Tests

The field test evaluated the effect of the inoculation of *ZmBA DSM7* on the maize yield. The experimental area was divided into 39 m^2^ plots, maize seeds were sown with a distance of about 20 cm.

In the black soil field, The experiment was performed in the experimental fields of Heilongjiang Academy of Agricultural Sciences (Longitude 126. 847941 Latitude: 45.840820). B426 were used for the open field tests in Haebin. *ZmBA DSM7* inoculation was performed by seed soaking and seedling root irrigation at two key growth stages, the four treatments were as follows: The first group maize for seed soaking CK (the control group was soaked in sterile water). The second group for maiz seed soaking, (maize seeds were immersed in a *ZmBA DSM7* suspension (10^8^ CFU·mL^−1^) for 12 h before sowing). The third group for seedling root irrigation at CK (no inoculation, irrigated with equal volume water) at the four-leaf stage; The fourth group for seedling root irrigation at *ZmBA DSM7* suspension (10^7^ CFU·mL^−1^) via root drenching. No chemical fertilizers were applied to avoid confounding effects on soil salinity and microbial activity.

In a two-year field experiment conducted in a saline soil area (soil electrical conductivity 9.6 dS·m^−1^, pH 8.8) in Daqing city, Longdan181 were used for the open field tests. The experiment was designed with two treatments: a non-inoculated control group (CK), a group of our-leaf stage maize seedling root irrigation at *ZmBA DSM7* suspension (10^7^ CFU·mL^−1^) via root drenching.

Yield-related traits were measured at the maturity stage: At maturity, ear characteristics (ear length, ear diameter, number of rows per ear, number of kernels per row) and grain yield (Measure the 100-kernel weight by weighing 3 samples of 100 kernels each and calculating the average) were measured.

The soil samples from 0–20 cm depth were collected to analyze changes in soil EC and pH before sowing and after harvest.

### 4.5. Statistical Analysis

Data were analyzed using one-way ANOVA, and means were compared with Duncan’s multiple range test (*p* < 0.05) using SPSS 23.0 software.

## 5. Conclusions

*Bacillus amyloliquefaciens ZmBA DSM7* is a highly effective, multi-talented PGP for conferring alkaline stress tolerance in maize. Enhance maize tolerance to alkali stresses by improving photosynthesis, membrane integrity, and osmotic adjustment, while also contributing to soil quality improvement. These findings highlight the potential of endophytes as bio-inoculants for sustainable agriculture in saline-alkali regions.

## Data Availability

Data will be made available on request.
